# Induction of HIV-1 Gag-specific memory T cells in Chacma baboons by MVA prime and VLP boost vaccine regimen

**DOI:** 10.1186/1742-4690-9-S2-P27

**Published:** 2012-09-13

**Authors:** V Singh, G Chege, E Shephard, A Williamson

**Affiliations:** 1 University of Cape Town, Cape Town, South Africa

## Background

We previously reported induction of HIV-specific responses in Chacma baboons following immunization with SAAVI MVA-C (MVA) and HIV-1 Pr55 Gag virus-like particles (VLPs) in a prime-boost vaccination strategy. In the current study, we characterised the vaccine specific memory T cells by flow cytometry.

## Methods

Peripheral blood mononuclear cells (PBMC) from baboons primed with MVA and boosted with VLPs (n=3) or vaccinated with VLPs only controls (n=2) were stimulated with HIV-1 Gag peptide pools. T cell cytokine production (multiplex TNF-a, IFN-y and IL-2) and memory phenotype was determined by flow cytometry. Human anti-CD28 and CD95 antibodies were used to delineate effector memory (Tem) and central memory (Tcm) T cells.

## Results

Vaccine specific memory responses were detectable one week after MVA prime. At peak T cell response (four weeks after VLP boost), the frequency of cytokine producing cells in prime-boost animals (mean response: 0.21%±0.012 and 0.242% ±0.049 of CD4+ and CD8+ cells respectively) was higher than in control animals (mean response: 0.066%±0.005 and 0.034%± 0.016 of CD4+ and CD8+ cells respectively). Gag-specific CD4+ cells from the prime-boost animals were significantly skewed towards a Tcm phenotype (>95%) of total cytokine responses compared to the Tem phenotype (<2%). A similar memory distribution profile of Gag-specific CD4+ cells was maintained 20 weeks after the VLP boost. At this time, Gag-specific CD8+ cells were evenly distributed between Tcm (~40%) and Tem (~60%) phenotypes. Vaccine specific memory responses were preserved 20 weeks after the VLPs boost (mean: 0.128%±0.025 and 0.147%± 0.039 of CD4+ and CD8+ cells respectively) in the prime-boost animals.

## Conclusion

In conclusion, the MVA prime and VLP boost induced Gag-specific cytokine producing Tcm and Tem defined by expression of CD28 and CD95. These cells were detected up to 20 weeks post vaccination suggesting these vaccines could be potential HIV-1 vaccine candidates.

